# Effects of *Saccharomyces cerevisiae* in association with *Torulaspora delbrueckii* on the aroma and amino acids in longan wines

**DOI:** 10.1002/fsn3.1076

**Published:** 2019-08-05

**Authors:** Kanokchan Sanoppa, Tzou‐Chi Huang, Ming‐Chang Wu

**Affiliations:** ^1^ Department of Food Science National Pingtung University of Science and Technology Pingtung Taiwan; ^2^ Department of Biological Science and Technology National Pingtung University of Science and Technology Pingtung Taiwan

**Keywords:** odor activity values, *Saccharomyces cerevisiae*, sequential, simultaneous, *Torulaspora delbrueckii*

## Abstract

This study investigated the effects of monocultures of *Saccharomyces cerevisiae* and *Torulaspora delbrueckii* as well as simultaneous and sequential cultures of *S. cerevisiae* and *T. delbrueckii* on the nonvolatile and volatile compounds in longan wines. The four cultures had similar characteristics in longan wines. The main amino acids in all the fermentations were glutamic acid, arginine, alanine, leucine, proline, and GABA. The main volatile compounds in longan wines were ethanol, isoamyl alcohol, isobutanol, 2‐phenylethanol, isoamyl acetate, ethyl decanoate, ethyl octanoate, ethyl hexanoate, and ethyl acetate, which can contribute more desired aroma compounds in wines. Among the four treatments, the longan wine fermented with the simultaneous culture produced the highest total volatile aroma content (345.26 mg/L). The simultaneous culture also had a better ability to generate a high level of the main volatile compounds in longan wines and also could achieve a noticeable intensity of floral and fruity aromas of wine as evaluated by calculation of the odor activity values.

## 1 INTRODUCTION

Longan (*Dimocarpus longan* Lour) is one of the main commercial fruits of Thailand, which can be consumed as fresh or processed, such as canned longan and dried longan. Fresh longan has high polyphenols, rich amount of sugar, potassium, copper, ascorbic acid, and other vitamins, and also has a relatively high content of amino acids, which favor its use as a raw material for wine production (Trinh, Yu, Curran, & Liu, 2012), while dried longan essentially consists of glucose, sucrose, fructose, and free amino acids as well as various vitamins and minerals. Various compounds have been found during the drying process of longan, such as ocimenes, furfural, isoamyl alcohol, linalool oxide, benzenemethanol, ethyl hexadecanoate, and 2‐furancarboxylic acid. The flavor of dried longan may be derived from a Maillard reaction and/or caramelization during the drying process (Chang, Chang, Yu, Lin, & Yen, 1998).

Nowadays, *Saccharomyces cerevisiae* strains are typically used for winemaking, which regulate fermentation in juices and are tolerant to acidic, alcoholic, anaerobic conditions, and high sugar (Chen & Liu, 2016). Recent studies have shown the using non‐*Saccharomyces* strains, which are considered to be good candidates for commercial winemaking as several have shown high oenological potential (Azzolini et al., 2012). *Torulaspora delbrueckii* strains are now available for commercial winemaking. *Torulaspora delbrueckii* strains can produce good oenological characteristics, such as a higher concentration of esters, higher alcohols, terpenes, and phenolic aldehydes as well as 2‐phenylethanol, linalool, methylvanilin than *S. cerevisiae*, together with good glycerol production, a lower production of volatile acidity, acetaldehyde, and acetoin (Taillandier, Lai, Julien‐Ortiz, & Brandam, 2014). However, this yeast strain is less tolerant to low available oxygen conditions than *S. cerevisiae* (Hansen, Nissen, Sommer, Nielsen, & Arneborg, 2001). Therefore, it would be interesting to study a coculture of *S. cerevisiae* and *T. delbrueckii*, especially as mixed inoculations can give higher ethyl hexanoate and 3‐hydroxybutanoate contents than a monoculture with *S. cerevisiae a*nd *T. delbrueckii*, as well as high contents of terpenes, phenylacetaldehyde, ethyl decanoate, ethyl butanoate, 3‐methyl‐1‐pentanol, 2‐phenylethanol, isoamyl acetate, and isobutyl acetate (Renault, Coulon, de Revel, Barbe, & Bely, 2015; Zhang, Luan, Duan, & Yan, 2018).

In spite of the studies described above, *T. delbrueckii* and cocultures have not been used in longan wine. Previous research on longan wines is limited, with most of the few studies in the literature focused on winemaking from fresh longan and process optimization, including functional activity (Liu et al., 2018). Therefore, using dried longan is an interesting option to produce wine due to dried longan containing a wide variety of nutrients, especially amino acids, that are suitable for culture with yeasts. Amino acids are the important precursors to volatile flavored compounds produced by yeasts, which play an important role in the quality of the wine (Trinh et al., 2012).

In this research, dried longan was used as a raw material for winemaking. Dried whole longan is a processed agricultural product, by which value has been added to longan fruit, which represents an important economic crop of Thailand. The aim of this research was to investigate the effects of mono‐, simultaneous, and sequential cultures of *S. cerevisiae* and *T. delbrueckii* on the nonvolatile and volatile compounds in longan wines. The contribution of fermentation metabolites by yeast to functional profile and the aromatic of wine is well documented; however, the biotechnological application of this knowledge is still rather limited to date. Understanding and modeling the relationship between the production of desirable compounds and nutrient availability by yeasts are the main objectives of this study. The research will provide insights into the characteristic flavor of longan wines and a scientific basis for further developing this industry.

## 2 MATERIALS AND METHODS

The major material utilized in this study was dried whole longan, purchased from a local market at Lamphun, Thailand, which was dried by a hot air dryer at 80°C for 17 hr, then at 75°C for 20 hr, then at 65°C for 10 hr, and finally stored at 4°C until analysis.

Potato dextrose agar and bacteriological peptone were purchased from Oxoid. dl‐Malic acid was purchased from Sigma‐Aldrich. Alanine, arginine, aspartic acid, gamma‐aminobutyric acid, glutamic acid, glycine, isoleucine, leucine, lysine, phenylalanine, proline, serine, threonine, tyrosine, and valine were supplied by Sigma Chemicals. AccQ‐Fluor reagent kits containing borate buffer, reagent powder, and acetonitrile were supplied by Waters Co. Ltd.

### 2.1 Yeast strains and culture media

*Saccharomyces cerevisiae* strain C12 L.A. *Bayanus* (Australia) and *T. delbrueckii* ATCC 20100; I AM 4383 (Taiwan) were used in this experiment. The cultured yeasts were incubated under static conditions at 25°C for up to 48 hr and finally stored at −80°C before use.

### 2.2 Pretreatment of the longan juice

First, 100 g of dried longan was placed into a flask and 300 ml of distilled water was added. The samples were blended with a blender until the samples were fined and then the longan juice was passed through a filter. Longan juice was obtained, the sugar content adjusted (°Brix) to 20.0% with pure sucrose and the pH to 3.5 with 50% w/v dl‐malic acid, and then sterilized with 100 ppm of potassium metabisulfite (Chen & Liu, 2014, 2016).

### 2.3 Fermentation of the longan juice

Fermentations of the sterile longan juices were carried out in triplicate in sterile Erlenmeyer flasks at 25°C under static conditions for 14 days. Four different fermentations were conducted, two with the monocultures and the two cocultures (simultaneous and sequential). A simultaneous culture was performed to inoculate *S. cerevisiae* and *T. delbrueckii* at the same time. A sequential culture was performed to inoculate *T. delbrueckii* 24 hr before *S. cerevisiae*. The monoculture and cocultures were inoculated with 1 × 10^6^ CFU/ml for both *S. cerevisiae* and *T. delbrueckii.*

### 2.4 Measurement of the physiochemical parameters

During the fermentation, samples were collected every 2 days for up to 14 days and were then stored at −20°C before use. The samples were analyzed for their pH value using a pH meter (Metrohm), °Brix value with a refractometer (REED), total sugar content using the phenol–sulfuric acid method, and total acidity content by titration.

### 2.5 Analysis of the amino acids

Analysis of the amino acids was performed based on the method developed by Water AccQ‐Tag (Waters, 1993) and as used according to Zeng et al. (2015). Chromatographic separation by reversed‐phase HPLC was carried out using a Hypersil GOLD™ Column C18 (4.6 mm × 150 mm, 3 µm; Thermo Fisher Scientific) equipped with a fluorescence detector (excitation, 250 nm; emission, 395 mm).

### 2.6 Analysis of the volatile compounds

The volatile compounds were analyzed using the headspace (HS) solid‐phase microextraction (SPME) method with a 30/50 µm DVB/Carboxen^™^/PDMS StableFlex^™^ fiber (Supelco: 57348‐U, Sigma‐Aldrich) coupled with a gas chromatography (GC) mass spectrometer (MS; HS‐SPME‐GC‐MS). Ten milliliters of longan wine was placed in a 20 ml headspace vial, and then 100 ppm octanol was added into the bottle as the internal standard, to which was added 3.5 g of sodium chloride. The program consisted of swirling the vial at 250 rpm for 10 min at 40°C, inserting the fiber to adsorb at 40°C for 10 min at 250 rpm, and then transferring the fiber for desorption at 230°C for 3 min.

GC‐MS analysis was performed with an Agilent 7890B, equipped with a DB‐Wax UI column (60 m × 0.25 mm coated with 0.25 µm film thickness). The operation conditions were according to Liu et al. (2018).

The odor activity values were calculated by dividing the known thresholds.

### 2.7 Statistical analysis

Statistical differences among wine samples inoculated with different yeast cultures were determined by ANOVA and Scheffe's test using the SPSS 20.0 software (SPSS Inc.) at *p* < 0.05. Principle component analysis (PCA) was performed on the volatile compounds using the Minitab 18 and SPSS 20.0 software.

## 3 RESULTS AND DISCUSSION

### 3.1 Yeast population and physiochemical parameters

**Figure 1:**
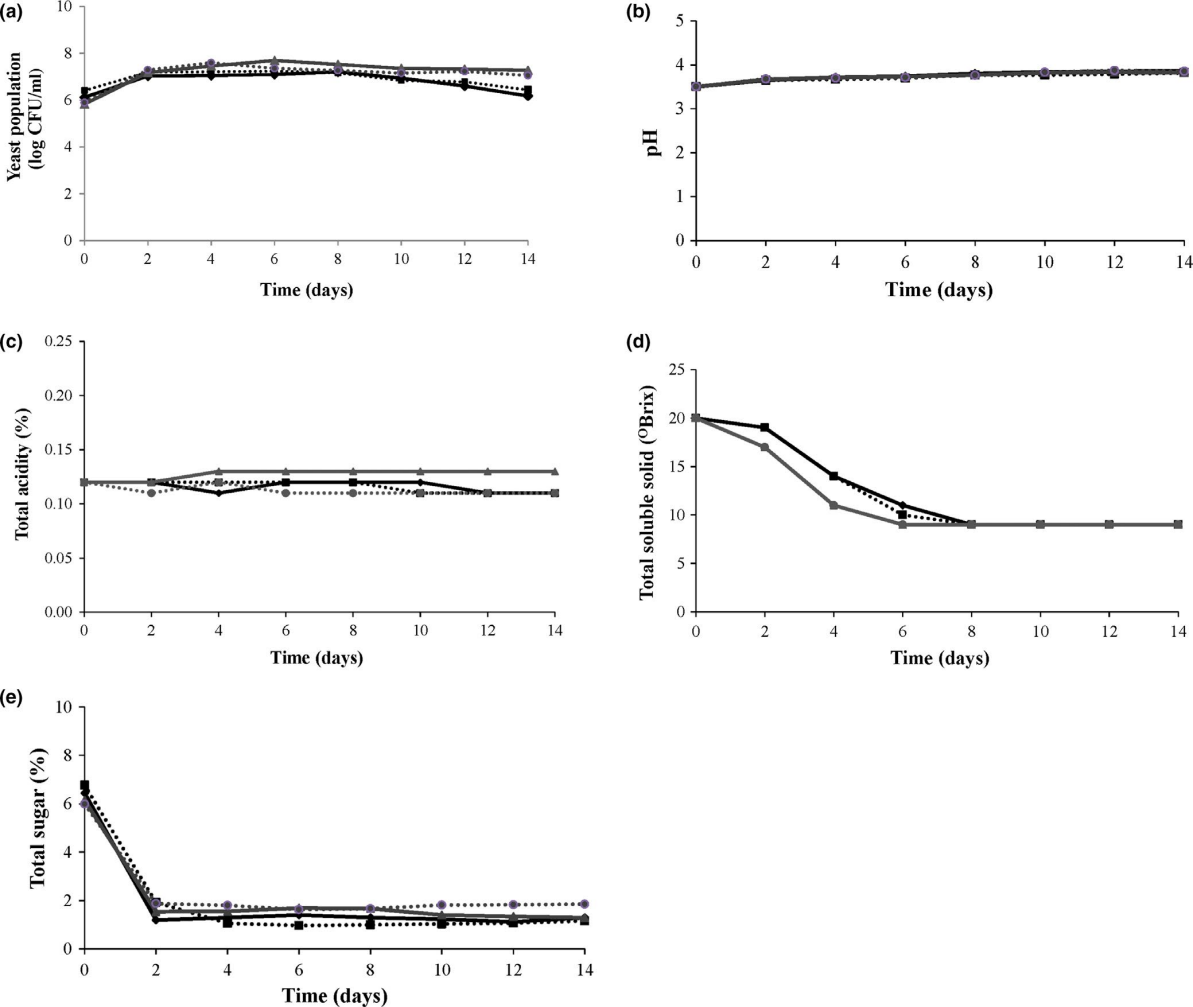
Changes in yeast population (CFU/ml) (a), pH (b), total acidity (%) (c), total soluble solid (°Brix) (d), and total sugar (%) (e) during fermentation from dried whole longan cultured with *Saccharomyces cerevisiae* (♦), *Torulaspora delbrueckii* (□)*,* simultaneous (▲), and sequential (●)

The results demonstrated that the initial measure of yeast cells of all the fermentations was 1 × 10^6^ CFU/ml (Figure 1a). The count of yeast cells decreased continuously as the wine fermentation time increased and as the yeast used nutrients in the juice during fermentation and converted them into alcohol and the compounds. All the fermentations increased rapidly during the first 2 days. The yeast populations reached the maximum number of 7.69 log CFU/ml at the 6th day in the simultaneous culture. After the 8th day, the yeast population in both the mono‐ and cocultures in wines decrease consistently, due to sugar from fruit juice being used up during the fermentation. Some previous research has reported that *T. delbrueckii* was more sensitive to low available oxygen conditions than *S. cerevisiae* and found that some toxins produced by *S. cerevisiae* inhibited the growth of *T. delbrueckii* strains (Hansen et al., 2001). Nevertheless, in our work, all the fermentations retained a high level of viability, both for the pure culture with *T. delbrueckii* and the coculture, as was also reported by Taillandier et al. (2014).

The four fermentations in these wines showed similar characteristics in terms of total acidity, pH, total sugar, and total soluble solids. The pH of all the wines increased moderately after fermentation around pH 3.5–3.9 (Figure 1b), while the total acidity during wine fermentation was relatively constant, ranging about 0.10%–0.13% (Figure 1c). The pH and total acidity values were correlated to each other. In our experiments, the pH value slightly changed. The total acidity did not fluctuate much during fermentation, similar to the result found by Trinh et al. (2012). There were some changes in total soluble solid during wine fermentation (Figure 1d). The Brix value decreased initially and then stabilized. The final Brix value was 9%. The total sugar of all wines in the fermentation decreased rapidly the first during 2 days of fermentation and then stabilized (Figure 1e).

### 3.2 Amino acid

Alanine and proline were the main amino acids found in longan juice, while threonine and lysine were not found in longan juice but were found in longan wines (Table 1). The results showed that the major amino acids in longan wines cultured with different yeast strains were glutamic acid, arginine, alanine, leucine, proline, and GABA. All the other amino acids in the wines were present in amounts that did not exceed 0.50 mg/100 ml. Proline was present in the highest amount in longan wines, similar to the results in lychee wines reported by Chen and Liu (2014). It is possible that proline is not a desirable nitrogen source and cannot be utilized in the same way as other amino acids under anaerobic conditions by yeast; however, proline may be an intermediate in the synthesis of glutamate or α‐keto glutarate and ammonium and can also be synthesized from glutamic acid (Chen & Liu, 2014). In the present research, the *S. cerevisiae* monoculture utilized the highest content of nitrogen, followed by *T. delbrueckii* culture, and then the simultaneous and sequential cultures, which were not significantly different to the results reported by Chen and Liu (2016), who found that the lowest amount of amino acids in lychee wines was from cultures with *S. cerevisiae* compared with different yeasts. Almost all the amino acids were consumed, except threonine and lysine.

**Table 1:** Amino acid concentrations of longan wines cultured with *Saccharomyces cerevisiae*, *Torulaspora delbrueckii,* simultaneous, and sequential

Amino acids (mg/100 ml)	Dried longan juice	Dried whole longan wines			
		*Saccharomyces cerevisiae*	*Torulaspora delbrueckii*	Simultaneous	Sequential
Asp	4.81 ± 0.05	ND	ND	ND	ND
Ser	1.76 ± 0.06^a^	0.34 ± 0.02^ab^	0.30 ± 0.01^b^	0.35 ± 0.01^ab^	0.37 ± 0.02^b^
Glu	8.06 ± 0.03^a^	0.75 ± 0.03^b^	0.78 ± 0.04^b^	0.75 ± 0.05^b^	0.81 ± 0.05^b^
Gly	0.64 ± 0.05^a^	0.28 ± 0.02^b^	0.34 ± 0.06^b^	0.34 ± 0.06^b^	0.36 ± 0.03^b^
Arg	4.70 ± 0.10^a^	0.62 ± 0.05^b^	0.58 ± 0.06^b^	0.65 ± 0.03^b^	0.65 ± 0.03^b^
Thr	ND	0.12 ± 0.01^NS^	0.11 ± 0.01^NS^	0.14 ± 0.05^NS^	0.15 ± 0.04^NS^
Ala	28.61 ± 1.18^a^	1.97 ± 0.03^b^	1.60 ± 0.05^b^	1.80 ± 0.06^b^	1.95 ± 0.03^b^
Pro	19.70 ± 0.31^a^	8.43 ± 0.04^d^	9.53 ± 0.08^b^	8.83 ± 0.09^c^	8.56 ± 0.05^d^
GABA	4.69 ± 0.33^a^	0.42 ± 0.05^b^	0.29 ± 0.04^b^	0.53 ± 0.05^b^	0.47 ± 0.04^b^
Tyr	1.27 ± 0.06^a^	0.26 ± 0.08^b^	0.26 ± 0.04^b^	0.30 ± 0.08^b^	0.30 ± 0.04^b^
Val	2.14 ± 0.14^a^	0.29 ± 0.09^b^	0.30 ± 0.05^b^	0.33 ± 0.04^b^	0.36 ± 0.03^b^
Lys	ND	0.22 ± 0.01^b^	0.24 ± 0.03^b^	0.22 ± 0.02^b^	0.30 ± 0.05^a^
Ile	0.61 ± 0.19^a^	0.14 ± 0.01^b^	0.08 ± 0.02^b^	0.15 ± 0.05^b^	0.15 ± 0.05^b^
Leu	2.69 ± 0.32^a^	0.53 ± 0.04^b^	0.47 ± 0.07^b^	0.59 ± 0.05^b^	0.62 ± 0.05^b^
Phe	0.64 ± 0.04^a^	0.27 ± 0.01^cd^	0.21 ± 0.01^d^	0.36 ± 0.05^b^	0.34 ± 0.05^b^

Abbreviations: ND, not detected; NS, not significant.

^a, b, c, d^ Statistical analysis ANOVA (*n* = 3) at 95% confidence level with same letters indicating no significant difference.

Amino acids are important nitrogen sources for yeast growth and fermentation activity. Their amount and composition affect the formation of volatile compounds that contribute to wine aroma (Zhang et al., 2018). Aromatic amino acids including branched‐chain amino acids are important precursors of flavor compounds. The catabolism of aromatic amino acids (tyrosine, tryptophan, phenylalanine) produces chemical and floral flavors, whereas branched‐chain amino acids (leucine, isoleucine, valine) can convert into compounds that contribute to fruity and sweet flavors (Ardo, 2006).

### 3.3 Volatile compounds

In total, 27 volatile compounds were detected in the longan wines (Table 2). The main volatile compounds in the wines were alcohols. Higher alcohols are secondary metabolites produced by yeast and can have both a positive and a negative impact on the aroma of the wine (Swiegers, Bartowsky, Henschke, & Pretorius, 2005). Ethanol was most abundant, ranging from 130 mg/L to 180 mg/L in wine fermentation. The ethanol content produced by *S. cerevisiae* was the highest in the wines, followed by *T. delbrueckii*, and then the simultaneous and sequential cocultures, respectively, indicating that *T. delbrueckii* had a lower fermentative activity than *S. cerevisiae,* and that the inoculation of *T. delbrueckii* in the pure culture leads to stuck fermentations (Chen & Liu, 2016; Renault et al., 2015). On the contrary, cocultures produce significantly higher concentrations of isoamyl alcohol and isobutanol than monocultures. Here, the simultaneous culture was the highest in 2‐phenylethanol, and similarly, Zhang et al. (2018) report that a coculture can contribute to variations in the higher alcohol profiles and concentrations in wines. Also, *T. delbrueckii* can release 2‐phenylethanol in a higher amount than *S. cerevisiae* (Renault et al., 2015). Isobutanol, isoamyl alcohol, and 2‐phenylethanol were synthesized in the yeast cell via the Ehrlich pathway. Isoamyl alcohol and isobutanol were derived from leucine and valine (Swiegers et al., 2005), while 2‐phenylethanol was derived from the phenylalanine (Etschmann et al., 2008). The catabolism process of branched‐chain amino acids involves a transamination to form α‐keto acid (α‐ketoisovaleric acid from valine, α‐keto‐β methylvaleric acid from isoleucine, and α‐ketoisocaproic acid from leucine), followed by a decarboxylation step, leading to the formation of aldehydes and the corresponding acids and alcohols (Swiegers et al., 2005).

**Table 2:** Volatile aroma contents (mg/L) in longan wines

Compounds	Dried longan juice	Dried whole longan wines			
		*Saccharomyces cerevisiae*	*Torulaspora delbrueckii*	Simultaneous	Sequential
Alcohols					
Ethanol	11.47 ± 4.06^d^	173.47 ± 1.80^a^	162.88 ± 7.98^b^	145.39 ± 4.61^c^	136.90 ± 5.26^c^
Isobutanol	ND	5.45 ± 0.09^a^	2.99 ± 0.23^b^	5.58 ± 0.44^a^	5.44 ± 0.70^a^
Isoamyl alcohol	ND	49.41 ± 0.90^b^	39.73 ± 3.76^c^	61.53 ± 3.29^a^	61.61 ± 3.96^a^
2‐Phenylethanol	ND	14.73 ± 0.06^NS^	15.51 ± 0.68^NS^	15.40 ± 0.55^NS^	14.76 ± 1.59^NS^
Sum	11.47	243.06	221.11	227.90	218.71
Esters					
Ethyl acetate	ND	4.98 ± 0.05^c^	6.06 ± 0.81^bc^	6.63 ± 0.94^b^	8.27 ± 0.43^a^
Ethyl benzoate	ND	0.70 ± 0.01^c^	0.75 ± 0.13^c^	1.57 ± 0.15^a^	1.02 ± 0.04^b^
Ethyl butanoate	ND	ND	ND	0.85 ± 0.03^NS^	0.90 ± 0.06^NS^
Ethyl decanoate	ND	7.82 ± 1.05^c^	15.89 ± 0.89^a^	16.04 ± 1.11^a^	10.27 ± 0.20^b^
Ethyl dodecanoate	ND	1.84 ± 0.10^c^	3.04 ± 0.23^b^	4.14 ± 0.15^a^	3.22 ± 0.17^b^
Ethyl heptanoate	ND	1.89 ± 0.07^c^	1.92 ± 0.07^c^	2.50 ± 0.06^a^	2.19 ± 0.07^b^
Ethyl hexanoate	ND	5.44 ± 0.05^c^	7.52 ± 1.39^b^	9.35 ± 0.85^a^	8.82 ± 0.74^ab^
Ethyl nonanoate	ND	1.28 ± 0.12^a^	1.30 ± 0.04^a^	0.94 ± 0.06^b^	0.75 ± 0.11^c^
Ethyl octanoate	ND	20.51 ± 0.39^b^	30.74 ± 2.65^a^	30.48 ± 1.92^a^	23.77 ± 1.17^b^
Ethyl pentanoate	ND	ND	ND	1.01 ± 0.13^NS^	0.66 ± 0.10^NS^
Isoamyl acetate	ND	11.27 ± 0.39^b^	11.88 ± 0.31^b^	13.91 ± 0.42^a^	13.14 ± 1.08^a^
Sum	ND	55.73	79.1	87.42	73.01
Miscellaneous					
1,3,6‐Octatriene, 3,7‐dimethyl‐, (E)‐	0.27 ± 0.04^d^	9.22 ± 0.41^c^	21.63 ± 1.64^a^	14.70 ± 0.52^b^	13.69 ± 0.58^b^
1,3,7‐Octatriene, 3,7‐dimethyl‐, (E)‐	ND	0.76 ± 0.05^b^	1.86 ± 0.13^a^	ND	ND
2,3‐Butanediol	0.36 ± 0.13	ND	ND	ND	ND
Acetic acid	2.74 ± 0.79^c^	8.56 ± 0.08^b^	8.53 ± 0.31^b^	11.24 ± 0.93^a^	9.65 ± 0.73^b^
Benzenecarboxylic acid	0.09 ± 0.01	ND	ND	ND	ND
d‐Limonene	ND	ND	ND	0.80 ± 0.06	ND
Isovaleraldehyde	1.05 ± 0.43	ND	ND	ND	ND
Linalool	ND	1.06 ± 0.11^b^	1.24 ± 0.15^b^	1.44 ± 0.07^a^	1.58 ± 0.04^a^
Oxime‐, methoxy‐phenyl‐	ND	2.26 ± 0.09^NS^	2.47 ± 0.07^NS^	ND	ND
Sum	4.51	23.91	38.40	29.94	27.58
Total	27.45	322.70	338.61	345.26	319.30

Abbreviations: ND, not detected; NS, not significant.

^a, b, c, d^ Statistical analysis ANOVA (*n* = 3) at 95% confidence level with same letters indicating no significant difference.

Esters were the second most abundant volatiles in our wines. Esters provide fruity flavors and have a potential impact on the aroma profile of many commercially available wines (Swiegers et al., 2005). High concentrations of esters, such as ethyl octanoate, isoamyl acetate, ethyl decanoate, ethyl hexanoate and ethyl acetate, were found in longan wines (Table 2). Among the four treatments, the longan wine cultured simultaneously using *S. cerevisiae* and *T. delbrueckii* produced the highest total esters (87.42 mg/L).

The first group, that is, the acetate esters, are formed from acetyl‐CoA and alcohol (ethanol or higher alcohol derived from amino acid metabolism). The major acetate esters in longan wines found in this study were isoamyl acetate and ethyl acetate (Table 2). The concentrations of isoamyl acetate and ethyl acetate were higher in the cocultures than monoculture, in agreement with previous findings (Arslan, Celik, & Cabaroglu, 2018; Renault et al., 2015). The acetate esters were produced in much higher levels than ethyl ester; therefore, they impart significantly more influence over the flavor and fragrance (Saerens, Delvaux, Verstrepen, & Thevelein, 2010). The rate of acetate ester formation mainly depended on the concentration of alcohol and acetyl‐CoA and the activity of the alcohol acetyltransferase (AAT) (Zhang et al., 2014). The synthesis of acetate esters, such as ethyl acetate and isoamyl acetate, in *S. cerevisiae* was ascribed to at least three acetyltransferase activities: AATase, ethanol acetyltransferase, and isoamyl AAT (Lilly, Lambrechts, & Pretorius, 2000).

*Torulaspora delbrueckii* and the cocultures (simultaneous and sequential) produced a high amount of ethyl acetate. Acetyl‐CoA is the precursor for fatty acids and acetate ester biosynthesis (Chen & Liu, 2016). Under the fermentation conditions with a limiting amount of oxygen (coculture), medium‐chain fatty acyl‐CoAs accumulate, which results in increased acetate ester synthesis (Äyräpää & Lindström, 1977). In addition, the regular acetyl‐CoA‐consuming pathways are mostly inactive, leading to an accumulation of acetyl‐CoA in *S. cerevisiae*. Ester synthesis could therefore be enhanced (Malcorps & Dofour, 1992). The high concentration of acetate esters could be linked to oxygen depletion in the medium, induced by the presence of both yeasts in the medium.

The second group, ethyl esters with pleasant odors, are synthesized from an alcohol (ethanol) and a short‐ or medium‐chain fatty acyl‐CoA derivative. The biosynthesis of fatty acid ethyl esters follows the alcoholysis mechanism. Alcoholysis is essentially a transferase catalyzing reaction in which the acyl moiety of acyl‐CoA is transferred to an alcohol (Trinh et al., 2012). In the fatty acid synthesis, the saturated fatty acyl chain is extended by two carbon units during each cycle, starting from the addition of acetyl‐CoA onto malonyl‐CoA by acetyl‐CoA carboxylase. The acetyl‐CoA unit (C2) is thus expanded to a butyryl group (C4). The following cycles give rise to a hexanoyl (C6) and then an octanoyl (C8) group, which would be continued to a length of 10 carbons (Chan & Vogel, 2010).

The ethyl esters in longan wines were ethyl benzoate, ethyl butanoate, ethyl decanoate, ethyl dodecanoate, ethyl heptanoate, ethyl hexanoate, ethyl nonanoate, ethyl octanoate, and ethyl pentanoate (Table 2). The amounts of ethyl benzoate, ethyl dodecanoate, ethyl heptanoate, and ethyl hexanoate in the simultaneous culture were significantly higher than the others, while ethyl butanoate and ethyl pentanoate were found only in the cocultures. The concentration of ethyl ester depends on the concentration of the fatty acid precursor (Ilc, Werck‐Reichhart, & Navrot, 2016). Azzolini et al. (2012) reported that a significant amount of medium‐chain (C4–C10) fatty acid is accumulated in the *T. delbrueckii* culture. We postulated that ethyl esters in longan wines may be synthesized following the alcoholysis mechanism.

Others volatiles, including terpenes and acids, were detected in longan wines (Table 2). 1,3,6‐octatriene, 3,7‐dimethyl‐, (E)‐ had higher contents in the *T. delbrueckii* fermentation than others, while 1,3,7‐octatriene,3,7‐dimethyl‐, (E)‐ was found only with the pure *S. cerevisiae* and *T. delbrueckii*. Linalool is a monoterpene compound and is important to the aroma and flavor of wine (Swiegers et al., 2005). The simultaneous and sequential cultures presented a significantly higher concentration of linalool. Moreover, limonene is one of the most important compounds in the flavor and fragrance industries. Limonene was found only in the simultaneous culture.

### 3.4 Odor activity values (OAVs) of selected volatile compounds and principal component analysis (PCA)

Although about 27 volatiles compounds (Table 2) were identified in each wine, not all the compounds had a great impact on the overall odor of wines. Odor activity values were calculated as the ratio between the measured quantitative concentration of a substance in wine and its odor threshold. Generally, only the volatile compounds with an OAVs higher than 1 were considered to contribute to wine aroma (Chen & Liu, 2014). Ethyl octanoate can generate the highest OAVs, followed by ethyl hexanoate, isoamyl acetate, ethyl decanoate, isoamyl alcohol, 2‐phenylethanol, ethyl acetate, and isobutanol, respectively (Table 3). The results were consistent with previous studies reporting that in the lychee wines, ethyl hexanoate and ethyl octanoate had higher OAVs (Chen & Liu, 2014) and also Chen and Liu (2016) found that ethyl octanoate and ethyl hexanoate were the main compounds in the four monovarietal wines due to their high OAVs. These were responsible for the floral and fruity aromas in longan wines. In the alcohol group (except for ethanol), 2‐phenylethanol and isoamyl alcohol had OAVs higher than 1, which mean they could contribute fruity and rose‐like aromas. The highest OAVs of ethyl octanoate were 15,370.00 with *T. delbrueckii* culture, but with no significant differences among the simultaneous culture. Ethyl hexanoate had a significantly higher OAVs in the simultaneous culture of 1,870.00. Isoamyl acetate can contribute fruity and banana‐like aromas, and had a significantly higher OAVs in the wines with the simultaneous culture, for a value of 463.67, but there was no significant difference with the sequential culture.

**Table 3:** The concentration of main volatile compounds (mg/L) and odor activity values (OAVs) in longan wines cultured with different yeast strains

Compounds	Dried whole longan wines								Odor threshold value (mg/L)	Odor description
	*Saccharomyces cerevisiae*		*Torulaspora delbrueckii*		Simultaneous		Sequential			
	Concentration	OAV	Concentration	OAV	Concentration	OAV	Concentration	OAV		
Ethanol	173.47 ± 1.80^a^	–	162.88 ± 7.98^b^	–	145.39 ± 4.61^c^	–	136.90 ± 5.26^c^	–	–	Alcoholic
Isobutanol	5.45 ± 0.09^a^	0.14	2.99 ± 0.23^b^	0.07	5.58 ± 0.44^a^	0.14	5.44 ± 0.70^a^	0.14	40*	Fruity, banana
Isoamyl alcohol	49.41 ± 0.90^b^	1.65	39.73 ± 3.76^c^	1.32	61.53 ± 3.29^a^	2.05	61.61 ± 3.96^a^	2.05	30*	Banana, cheese, sweet
2‐Phenylethanol	14.73 ± 0.06^NS^	1.47	15.51 ± 0.68^NS^	1.55	15.40 ± 0.55^NS^	1.54	14.76 ± 1.59^NS^	1.48	10*	Floral, rose, sweet
Ethyl acetate	4.98 ± 0.05^c^	0.66	6.06 ± 0.81^c^	0.81	6.63 ± 0.94^b^	0.88	8.27 ± 0.43^a^	1.10	7.5*	Fruity, pineapple, sweet
Ethyl decanoate	7.82 ± 1.05^c^	39.10	15.89 ± 0.89^a^	79.45	16.04 ± 1.11^a^	80.20	10.27 ± 0.20^b^	51.35	0.2**	Fruity, floral, waxy
Ethyl hexanoate	5.44 ± 0.05^c^	1,088.00	7.52 ± 1.39^b^	1,504.00	9.35 ± 0.85^a^	1,870.00	8.82 ± 0.74^ab^	1,784.00	0.005**	Fruity, apple, strawberry
Ethyl octanoate	20.51 ± 0.39^b^	10,255.00	30.74 ± 2.65^a^	15,370.00	30.48 ± 1.92^a^	15,240.00	23.77 ± 1.17^b^	11,885.00	0.002*	Fruity, floral, pineapple
Isoamyl acetate	11.27 ± 0.39^b^	375.67	11.88 ± 0.31^b^	396.00	13.91 ± 0.42^a^	463.67	13.14 ± 1.08^a^	438.00	0.03***	Fruity, banana, sweet

^a, b, c^ Statistical analysis ANOVA (*n* = 3) at 95% confidence level with same letters indicating no significant difference.

* Guth (1997).

** Chen and Liu (2014).

*** Chen and Liu (2016).

**Figure 2:**
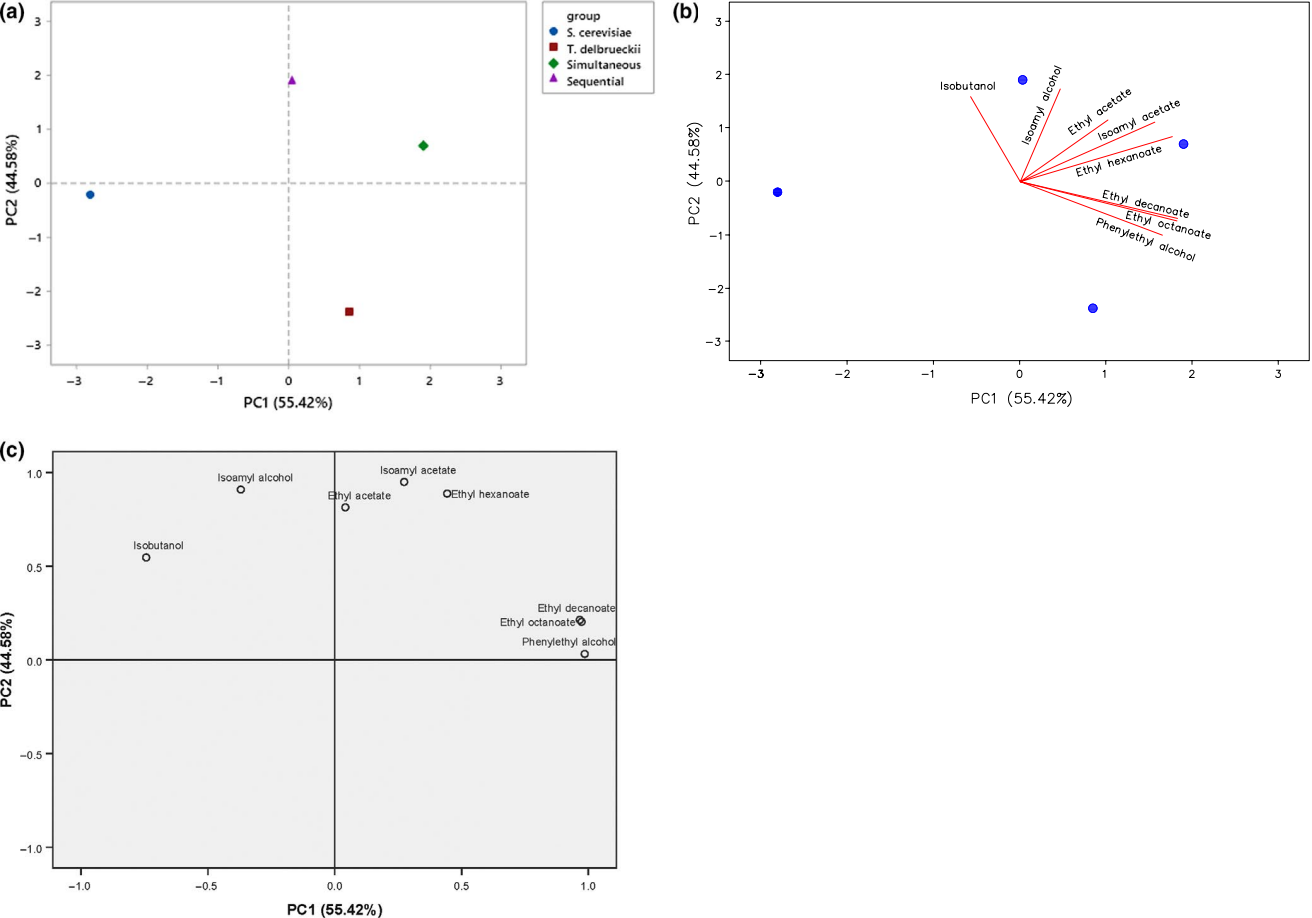
Principal component analysis (PCA) biplots of longan wines resulting from the selected volatile compounds and longan wines fermented with four cultures. (a) Score plots of yeast strains. (b) Biplot plots between yeast strains and volatile compounds. (c) Component plots of volatile compounds

PCA was applied to the volatile compound data to distinguish between the wines and identify the correlation between the yeast strains and aromatic compounds. The first and second principal components accounted for 92.34% of the total variance in the wines, with PC1 accounting for 55.42% of the total variance and PC2 44.58% (Figure 2). *S. cerevisiae* cultures could be clearly separated from *T. delbrueckii* cultures, possibly due to the different formation path of the aroma compounds in these two cultures (Figure 2a). Longan wines fermented with *S. cerevisiae* were in the negative part of PC1, and showed lower OAVs in wines. This characteristic leads to the diversity of simultaneous and sequential cultures in wines (Zhang et al., 2018). Simultaneous and sequential cultures in wines were positioned on the positive part of PC1 and PC2, suggesting that these cultures had a higher aromatic concentration. Isoamyl alcohol, isoamyl acetate, ethyl acetate, and ethyl hexanoate showed a higher correlation with the cocultures than the other compounds, which was consistent with the higher concentration of OAVs found in the cocultures (Figure 2b; Table 3). These results were consistent with previous studies that a coculture of *S. cerevisiae* and *T. delbrueckii* was a better inoculation method to generate more of the desired aroma compounds (Taillandier et al., 2014; Zhang et al., 2018). The component plots of the aroma compounds showed that ethyl hexanoate, ethyl octanoate, ethyl decanoate, ethyl acetate, 2‐phenylethanol, and isoamyl acetate were positioned on the positive part of both PC1 and PC2, suggesting that these volatile compounds could contribute more of the desired aroma compounds in wines (Figure 2c).

**Figure 3:**
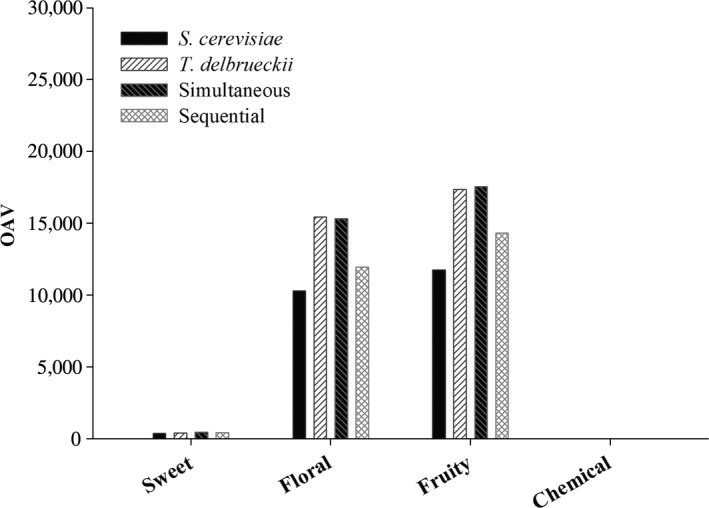
Aroma groups in longan wines cultured with *Saccharomyces cerevisiae, Torulaspora delbrueckii,* simultaneous, and sequential. Sweet group (phenylethyl alcohol, isoamyl alcohol, isoamyl acetate, and ethyl acetate), floral group (phenylethyl alcohol, ethyl decanoate, and ethyl octanoate), fruity group (isobutanol, isoamyl alcohol, ethyl acetate, ethyl decanoate, ethyl hexanoate, ethyl octanoate, and isoamyl acetate), chemical group (isoamyl alcohol, ethyl acetate, and isobutanol)

Volatile compounds in longan wines can be defined as a group of aromatic compounds with a similar odor description. Related compounds with an OAVs higher than 1 and with a similar odor description were classified into four groups, namely sweet, floral, fruity, and chemical. The fruity group had the highest odor description in the wines, followed by the floral, sweet, and chemical groups (Figure 3). The simultaneous culture achieved the highest amount of fruity, sweet, and chemical aromas, which may be due to the simultaneous culture producing higher contents of ethyl hexanoate and isoamyl acetate, while the *T. delbrueckii* monoculture had the highest amount of floral group, because this culture had higher 2‐phenylethanol and ethyl octanoate contents. The chemical group had a lower concentration than 10, which means it is not shown in Figure 3.

## 4 CONCLUSION

In this study, the effects of mono‐, simultaneous, and sequential cultures of *S. cerevisiae* and *T. delbrueckii* on the nonvolatile and volatile compounds in dried longan were investigated. The main amino acids in wines cultured with different yeast strains were glutamic acid, arginine, alanine, leucine, proline, and GABA. The main volatile compounds in longan wines were ethanol, isoamyl alcohol, isobutanol, 2‐phenylethanol, ethyl octanoate, ethyl decanoate, isoamyl acetate, ethyl hexanoate, and ethyl acetate, which could contribute more desired aroma compounds in wines. Longan wines with the simultaneous culture produced the highest total volatile aroma contents (345.26 mg/L) and total esters (87.42 mg/L). The amount of ethyl hexanoate, ethyl dodecanoate, ethyl heptanoate, and ethyl benzoate in the simultaneous culture had higher contents than in the other cultures, while ethyl butanoate and ethyl pentanoate were found only fermented by the cocultures. The simultaneous culture had the highest content of volatile compounds in longan wines, which had higher OAVs in the main volatile compounds in longan wines, as has been confirmed by several authors, who reported that cocultures can produce more higher esters that contribute to the aroma in wine.

## CONFLICT OF INTEREST

The authors declare no conflict of interest.

## ETHICAL STATEMENT

The study did not involve any human or animal testing.
